# Healthy aging affects cerebrovascular reactivity and pressure-flow responses, but not neurovascular coupling: A cross-sectional study

**DOI:** 10.1371/journal.pone.0217082

**Published:** 2019-05-16

**Authors:** Kayla B. Stefanidis, Christopher D. Askew, Timo Klein, Jim Lagopoulos, Mathew J. Summers

**Affiliations:** 1 Sunshine Coast Mind and Neuroscience–Thompson Institute, University of the Sunshine Coast, Birtinya, Qld, Australia; 2 School of Health & Sport Sciences, University of the Sunshine Coast, Sippy Downs, Australia; Ehime University Graduate School of Medicine, JAPAN

## Abstract

**Background and purpose:**

Aging leads to alterations in cerebrovascular function, and these are thought to contribute to cognitive decline/dementia. Disturbances to cerebral blood flow regulation have been reported, but the findings are inconsistent and to date no study has comprehensively tested the collective and independent contribution of these parameters in the same age range. Such lines of enquiry are vital since aging is a heterogeneous and complex process, with cerebrovascular parameters being differentially affected depending on the individual. A multicomponent comprehensive measure of cerebrovascular function, which accounts for such diversity, is needed to differentiate between healthy young and old adults.

**Methods:**

We tested the effect of aging on cerebrovascular function by comparing healthy young adults aged 18–30 and older adults aged 60–75, without cognitive impairments. Cerebrovascular blood flow velocity was assessed using transcranial Doppler ultrasound. Parameters included resting middle cerebral artery velocity (MCAv), neurovascular coupling, cerebrovascular reactivity to CO_2_ (hypercapnia and hypocapnia), and the pressure-flow response during a sit-to-stand procedure.

**Results:**

MANOVA revealed that collectively, the parameters discriminated the groups (*p* < .001). MCAv and pressure-flow responses were lower in the older group (*p* < .001). While there were no differences in hypercapnic responses (*p* = .908) and neurovascular coupling (*p* = .517), hypocapnic responses were elevated in the old (*p* = .002).

**Conclusions:**

Collectively, cerebrovascular parameters can distinguish between healthy young and older adults, with aging leading to reductions in MCAv, and altering cerebrovascular reactivity and pressure-flow responses under hypotensive conditions.

## Introduction

Prior research implicates cerebrovascular disease in the development of age-related cognitive decline and dementia, but the underlying vascular mechanisms are not well understood [1]. An improved understanding of the nature of normal cerebrovascular aging is needed to help establish the role that vascular dysfunction might play in cognitive decline and dementia.

Transcranial Doppler (TCD) ultrasound enables measurement of beat-to-beat blood flow velocity of major cerebral arteries and is commonly utilized for assessing the regulatory parameters of the cerebral circulation, including neurovascular coupling (NVC), cerebrovascular reactivity (responses to hyper- and hypocapnia) and the pressure-flow response (as a measure of cerebral autoregulation) [2] (See Table 1).

**Table 1:** Regulatory parameters of the cerebral circulation.

**Neurovascular coupling**	Neurovascular coupling (NVC) concerns the relationship between the cerebral vasculature and neural stimulation resulting in a region-specific change in CBF in response to a given neural demand [2].
**Cerebrovascular reactivity**	Cerebrovascular reactivity defines the capacity of the cerebral vessels to dilate and constrict in response to a vasoactive stimulus, such as partial pressure of arterial carbon dioxide (P_ET_CO_2_). Vessel dilation occurs in response to elevated P_ET_CO_2_ (hypercapnia) with reduced P_ET_CO_2_ (hypocapnia) leading to vasoconstriction [2].
**Cerebral autoregulation (indexed by the pressure-flow response)**	Cerebral autoregulation constitutes the ability of the cerebral vasculature to combat changes in cerebral perfusion pressure, such that CBF remains constant [2].

Reduced middle cerebral artery velocity (MCAv) has been consistently reported in older adults [3, 4]. However, data concerning the regulatory parameters of the cerebral circulation are less consistent, with some studies reporting reduced neurovascular coupling [5] and cerebrovascular reactivity [5–7], while others have found no effect of age on cerebrovascular function [8–13]. Further, some studies have reported enhanced cerebrovascular responses with healthy aging [14, 15].

To date, the regulatory factors of cerebrovascular function have commonly been studied in isolation, which may contribute to the variance reported in the literature and our limited understanding of aging on cerebrovascular function [2]. Indeed, the aging process is heterogeneous and complex with the likelihood that cerebrovascular parameters will be differentially affected depending on the individual. A related issue is that few studies have examined the differential effects of aging on cerebrovascular reactivity to elevated and reduced P_ET_CO_2,_ which may offer important insights into the effect of aging on vasodilation and vasoconstriction, respectively. Indeed, prior work has shown age-related changes in reactivity under hypercapnic conditions [4, 5]. A recent study by Galvin, Celi [14] measured hypercapnic and hypocapnic responses separately, and described greater hypocapnic responses in healthy older adults with and without coronary artery disease. Combined, these findings suggest that both responses warrant investigation in understanding the effects of aging of cerebrovascular reactivity to CO_2_.

Therefore, to effectively distinguish between young and old adults and in order to fully understand the nature of cerebrovascular aging, a multicomponent measure of cerebrovascular function (which captures all potential changes) is needed. Such knowledge is vital since a full understanding of the nature of cerebrovascular aging is an important step towards understanding the vascular bases of age-related cognitive decline and dementia, and can ultimately inform the development of intervention and prevention programs tailored to these aging populations. Therefore, the purpose of this study was to provide a more complete investigation of the independent parameters of cerebrovascular function, by examining both their collective and independent contribution in a sample of healthy young adults aged 18–30 years compared with older adults without cognitive impairments aged 60–75 years.

## Methods

### Participants

Twenty-nine healthy young adults aged 18–30 years (*Mean* = 23, *SD* = 4) and twenty-nine healthy older adults aged 60–75 years (*Mean* = 68, *SD* = 3) were recruited from the community of the Sunshine Coast in Australia. A total of 8 participants were excluded due to poor ultrasound insonation windows (n = 6), claustrophobia (n = 1) and non-attendance (n = 1). Exclusion criteria included documented current or previous history of heart disease or atherosclerotic cardiovascular disease (e.g., coronary artery disease, myocardial infarction, peripheral artery disease, carotid stenosis); stroke or transient ischemic attack (TIA); neurological or neurodegenerative disease (e.g., Parkinson’s disease, multiple sclerosis, dementia or mild cognitive impairment); epilepsy or traumatic brain injury; orthostatic hypotension; significant psychiatric illness including schizophrenia and bipolar disorder; drug and alcohol abuse; and uncorrected hearing or visual impediments. Current smokers, left-handers, persons with clinical anxiety/depression or uncontrolled hypertension/hypercholesterolemia and diabetics were also excluded.

Those meeting eligibility criteria attended an information session during which written informed consent was obtained and a medical history form was completed. The study was approved by the University of the Sunshine Coast Human Research Ethics Committee (S/17/1009) in accordance with the National Health and Medical Research Council (NHMRC) of Australia ethical guidelines and complied with the *Declaration of Helinski*.

## Materials & procedures

Participants were instructed to abstain from caffeinated food and beverages, nicotine and alcohol 12 hours prior to their assessment. In addition, they were asked to avoid vigorous exercise on the day. Room temperature was controlled at approximately 23 degrees Celsius. Height (cm) and weight (kg) measurements were recorded. Blood pressure of the brachial artery was measured manually on 2 occasions.

**Fig 1:**
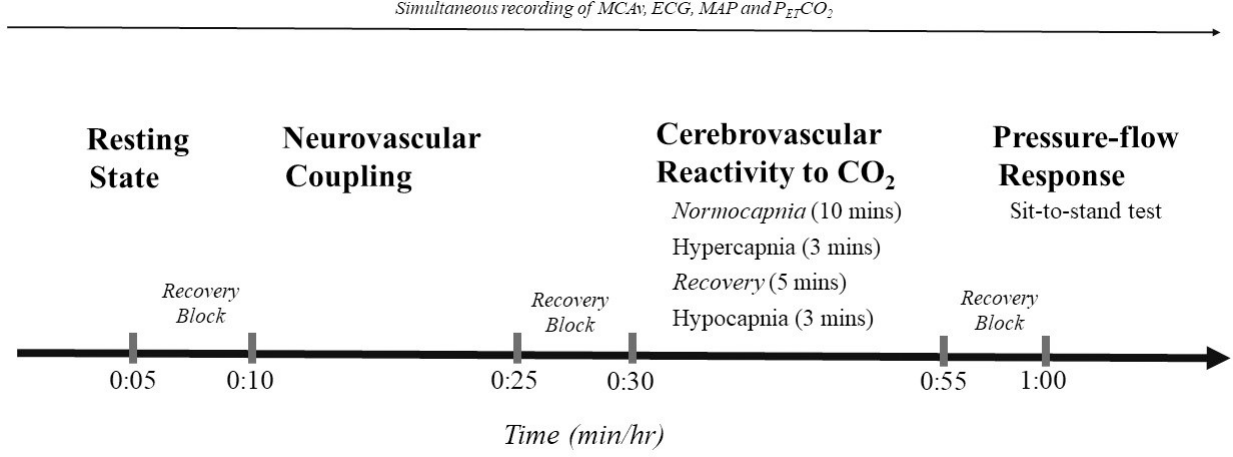
Cerebrovascular assessment timeline. MCAv: cerebral blood flow velocity of the middle cerebral artery. ECG: electrocardiography. MAP: mean arterial pressure. P_ET_CO_2_: partial pressure of arterial carbon dioxide.

Participants underwent a 2-hour assessment of their cerebrovascular function. Tasks were separated by 5-minute blocks to allow recovery and prevent cross-over effects (Fig 1).

Participants were instrumented for the continuous assessment of MCAv, heart rate, blood pressure and partial pressure of arterial carbon dioxide (P_ET_CO_2)._ Three electrocardiogram (ECG) electrodes were attached to the torso using a lead-II configuration for the assessment of heart rate. Blood pressure was continuously monitored at the index finger of the left hand via plethysmography, with a sling holding the hand at heart level (Finapress, Amsterdam, the Netherlands). MCAv was measured using transcranial Doppler ultrasonography (TCD, Multigon, Neurovision, Elmsford, N.Y., USA), where a 2MHz probe was secured over the transtemporal window above the zygomatic arch [as per standardised techniques, 2] with a padded headframe. Insonation depth began at 50mm and was adjusted as necessary to achieve an optimal signal. MCAv was assessed on left side, except in 1 participant where the right side was used to obtain an optimal signal. Expired CO_2_ concentration was monitored throughout using a fitted facemask and was used to calculate the partial pressure of end-tidal CO_2_ (P_ET_CO_2_) (Respironics, AD Instruments, Sydney, Australia). All data were continuously sampled at 1KHz and synchronized using a data acquisition system for offline analysis (Powerlab, ADInstruments, Sydney, Australia).

### Resting state

Once optimal signals were obtained, participants remained seated upright with their eyes open for 5 minutes. Mean MCAv was averaged over the final 30s.

### Neurovascular coupling

The NVC task commenced with 2 minutes rest with the eyes closed, then 2 minutes of silent reading, followed by 10 cycles of 20-seconds rest and 40 seconds reading. Maximum mean MCAv values during reading and minimum mean MCAv values during resting were averaged over the 10 cycles. The NVC index (NVC*i*) was calculated as the % maximum increase in MCAv during reading, relative to the minimum mean value during rest [NVC*i =* ((*maximum mean MCAv during reading–minimum mean MCAv at rest) / minimum mean MCAv at rest*) * 100] [16].

### Cerebrovascular reactivity to CO_2_

*Hypercapnia*. Participants initially breathed room air for 10 minutes (normocapnia). A gas mixture containing 5% CO_2_ (21% O_2_ in N_2_) was then inhaled for 3 minutes. Respiration rate was maintained at 16 breaths per minute using a metronome.

*Hypocapnia*. Participants hyperventilated voluntarily to maintain a threshold of approximately 20-25mmHg of P_ET_CO_2_ for 3 minutes. Respiration rates started at 25 breaths per minute and were adjusted as necessary. A 30-second block, where P_ET_CO_2_ values were maintained within the threshold, was used for analysis. Changes in MCAv relative to changes in P_ET_CO_2_ from (a) baseline to hypercapnia and (b) baseline to hypocapnia were calculated and expressed as %ΔMCAv/mmHgΔP_ET_CO_2_ [9].

### Pressure-flow response

A sit-to-stand test was used to measure changes in MCAv in response to changes in MAP. Repeated sit-to-stand maneuvers were performed at a frequency of 0.05Hz (sitting and standing every 10 seconds) for 5 minutes. Pressure-flow responses were calculated using the averaged maximum mean values during sitting and the minimum mean values during standing, expressed as: %ΔMCAv /%ΔMAP [17]. The advantage of using this approach was that it enabled the direction of change in MAP (in this case reductions in MAP from sit to stand), to be examined.

### Statistical analysis

The data were analysed using IBM SPSS Statistics 24 for Windows. Independent samples t tests and chi-square analyses were performed to test whether groups differed in baseline characteristics. Multivariate Analysis of Variance (MANOVA) was performed to determine whether the cerebrovascular parameters discriminated the old from the young both collectively and independently, while controlling for type I error inflation. Parameters of cerebrovascular function included resting MCAv, NVC*i*, as well as hypercapnic, hypocapnic and pressure-flow responses. Partial eta squared (η^2^) was used to quantify the magnitude of effects, with .01 representing a small effect, .09 a medium effect and .25 a large effect [18]. Where data were not available due to technical difficulties (<11%), the group mean was used [19]. Little’s MCAR test confirmed these data were missing at random, *p* = .520.

## Results

Groups did not differ significantly based on BMI or physical activity levels and diastolic and systolic BP was significantly higher in the old (see Table 2). Gosling’s Pulsatility Index (PI) at rest indicated no difference between the young (*M* = .90, *SD* = .09) and old group (*M* = .93, *SD* = .14) in vascular resistance, *p* = .280.

**Table 2:** Baseline characteristics.

Baseline Characteristics	Young *n = 29*	Old *n* = 29	P value
Age (years)	23 (4)	68 (3)	.000
Sex n (% female)	17 (59%)	12 (41%)	.189
BMI	24.41 (4.03)	26.44 (4.55)	.077
Physical Activity Levels (Total MET)	4742.33 (4205.1)	5365.43 (5102.01)	.630
Systolic BP mmHg	112 (12)	123 (16)	.005
Diastolic BP mmHg	63 (8)	70 (10)	.009
Medication use (n, %)			
BP Lowering	0	5 (17%)	.019
Lipid regulating	0	8 (28%)	.002
Antiplatelet (aspirin)	0	1 (3%)	.313

*Note*: Values expressed as Mean (SD) unless otherwise indicated. BMI: Body Mass Index (weight kg/height m^2^), Total MET: metabolic equivalents MET-minutes/week.

When combined, the cerebrovascular parameters distinguished the old from the young, accounting for 60% of the variance (Wilk’s lambda .397, *F*(5,52) = 15.78, *p* < .001, η^2^_p_ = .603, power = 1.00). The significant MANOVA was followed-up by between-subject contrasts to determine which factors independently discriminated between groups. These revealed that resting MCAv and pressure-flow responses were significantly reduced in the old, exhibiting large magnitude effects: η^2^_p_ = .353 and .378, respectively. In addition, responses to hypocapnia were significantly elevated in the old relative to the young. This was a medium to large effect, η^2^_p_ = .159. No significant differences in NVC*i* or hypercapnic responses were observed (η^2^_p_ = .008 and .00) (see Table 3).

**Table 3:** Cerebrovascular parameters.

Parameters	Young	Old	*F* value	*P* value	η^2^_p_	Power
	*Mean (SD)*	*Mean (SD)*				
Resting MCAv cm s^–1^	64.97 (8.34)	51.22 (10.47)	30.59	.000	.353	1.00
NVCi %ΔMCAv	21.61 (6.83)	22.90 (8.19)	.43	.517	.008	.098
Hypercapnia						
Baseline	30.88 (2.74)	30.32 (2.78)		.461		
%ΔMCAv/ΔP_ET_CO_2_	2.91 (1.25)	2.95 (1.49)	.01	.908	.000	.051
Hypocapnia						
Baseline	30.67 (2.31)	29.52 (2.51)		.092		
%ΔMCAv/ΔP_ET_CO_2_	1.12 (1.12)	2.19 (1.38)	10.60	.002	.159	.892
Pressure-flow %ΔMCAv/%ΔMAP	.91 (.24)	1.30 (.27)	34.03	.000	.378	1.00

*Note*: MCAv: mean cerebral artery blood flow velocity, NVC*i*: neurovascular coupling index, P_ET_CO_2:_ partial pressure of arterial carbon dioxide, MAP: mean arterial pressure.

To confirm that medication use (i.e., BP lowering, lipid regulating and antiplatelet (aspirin)) did not moderate effects, a second MANOVA was performed excluding such cases. This did not affect the results (Wilk’s lambda .408, *F*(5,41) = 11.90, *p* < .001, η^2^_p_ = .592, power = 1.00), such that differences in MCA (*p <* .001), hypocapnic responses (*p* = .026) and pressure-flow responses (*p* < .001) remained statistically significant.

## Discussion

The results of this study indicate that a multicomponent assessment of cerebrovascular parameters discriminated between the young and old group. This finding is novel and meaningfully significant. Given that the aging process is heterogeneous with a high degree of inter-individual variability, a measure of cerebrovascular function that encapsulates the range of variance within a population is essential to examine the nature of cerebrovascular aging.

Prior research has reported a reduction in CBF with increasing age [3, 4], but studies investigating regulatory parameters of cerebrovascular function have yielded inconsistent results. In contrast to other studies investigating autoregulatory pressure-flow responses using TCD [10, 12], we found greater reductions in MCAv in response to decreases in MAP (hypotension) in the old compared with the young. This is particularly noteworthy since aging is already associated with reductions in CBF. Indeed, poor CBF regulation under hypotensive conditions may lead to cerebral hypoperfusion, ischemic injury and ultimately changes in cognitive function [20, 21]. In regard to NVC, recent studies have shown that NVC is unaffected in older adults [9, 22]. Our data corroborate findings from these studies, strengthening the notion that the neurovascular coupling is preserved in healthy aging.

Interestingly, we identified differential effects of aging on cerebrovascular reactivity to CO_2,_ such that hypercapnic responses were unaffected, but hypocapnic responses were significantly elevated in the old group. This data is consistent with Galvin, Celi [14] reporting no effect of aging on hypercapnia, but elevated hypocapnic responses in a small sample of older adults with and without coronary artery disease. Notably, no differences were found between the groups, suggesting that such changes may be attributable to aging.

Notably, it was the test of vasoconstriction, and not those of vasodilation (i.e., NVC and responses to hypercapnia) that differentiated the old from the young. Although the underlying mechanisms remain unclear, alterations in vascular tone may account for the present findings [14]. For instance, given that resting MCAv was reduced, predilatory compensatory mechanisms may have led to the elevated hypocapnic response observed in the old group [14, 23]. Indeed, such patterns warrant further investigation, but do highlight the importance of assessing all regulatory components concurrently. The use of this approach is a major strength of this study.

### Limitations of the present study

There are points to consider in interpreting the present findings. First, it is important to note that TCD does not provide an absolute measure of CBF and rests on the premise that the diameter of the vessel under measure does not change. Whilst the changes observed in MAP and P_ET_CO_2_ observed in the present study were unlikely to be of sufficient magnitude to have any effect [24, 25], we do acknowledge the potential for slight changes in diameter of the MCAv. Other points to mention is that these measures were not assessed bilaterally and data was lost due to poor insonation windows, artifact and claustrophobia, thereby reducing our sample size. In addition, we cannot ignore the possibility of underlying cerebrovascular disease in the old group since participants did not undergo formal medical screening for CVD and indeed some were on pharmaceuticals to control for hypertension and hypercholesterolemia (including BP lowering, aspirin and lipid lowering medications). Nonetheless it is important to emphasize that BP was in the normotensive range in the old group and excluding such participants from the analysis did not affect the results.

## Conclusions

In conclusion, the results of this study suggest that aging is associated with reductions in MCAv, may increase cerebrovascular reactivity under hypocapnic conditions and weaken the brain’s capacity to combat reductions in pressure. Importantly, the results suggest that a multicomponent assessment of cerebrovascular parameters can distinguish older adults from healthy young adults. Since aging is heterogeneous and complex, this multicomponent measure of cerebrovascular function (which encapsulates the wide spectrum of change in this population), may be invaluable in terms of differentiating between normal and pathological aging, as well as providing insights into potential mechanisms at play in the case of age-related and neurodegenerative disease.
